# N-terminus oligomerization is conserved in intracellular calcium release channels

**DOI:** 10.1042/BJ20131061

**Published:** 2014-03-28

**Authors:** Spyros Zissimopoulos, Jason Marsh, Laurence Stannard, Monika Seidel, F. Anthony Lai

**Affiliations:** *Wales Heart Research Institute, Cardiff University School of Medicine, Institute of Molecular and Experimental Medicine, Heath Park, Cardiff CF14 4XN, U.K.

**Keywords:** inositol 1,4,5-trisphosphate receptor, intracellular calcium release channel, N-terminus, ryanodine receptor, tetramerization, AD, activation domain, DNA-BD, DNA-binding domain, HA, haemagglutinin, HEK, human embryonic kidney, IP_3_, inositol 1,4,5-trisphosphate, IP_3_R, IP_3_ receptor, RyR, ryanodine receptor, SR, sarcoplasmic reticulum, Y2H, yeast two-hybrid

## Abstract

Oligomerization of all three mammalian ryanodine receptor isoforms, a structural requirement for normal intracellular Ca^2+^ release channel function, is displayed by the discrete N-terminal domain which assembles into homo- and hetero-tetramers. This is demonstrated in yeast, mammalian cells and native tissue by complementary yeast two-hybrid, chemical cross-linking and co-immunoprecipitation assays. The IP_3_ (inositol 1,4,5-trisphosphate) receptor N-terminus (residues 1–667) similarly exhibits tetrameric association as indicated by chemical cross-linking and co-immunoprecipitation assays. The presence of either a 15-residue splice insertion or of the cognate ligand IP_3_ did not affect tetramerization of the IP_3_ receptor N-terminus. Thus N-terminus tetramerization appears to be an essential intrinsic property that is conserved in both the ryanodine receptor and IP_3_ receptor families of mammalian intracellular Ca^2+^ release channels.

## Abstract 2

Intracellular Ca^2+^ channels are of paramount importance for numerous cellular processes. In the present paper we report on a novel N-terminus intersubunit interaction, an essential structure–function parameter, which is conserved in both families of intracellular Ca^2+^ channels.

## INTRODUCTION

RyRs (ryanodine receptors) and IP_3_Rs [IP_3_ (inositol 1,4,5-trisphosphate) receptors] are homologous membrane protein channel families that mediate the acute Ca^2+^ release from internal stores essential for the activation of numerous physiological processes [1,2]. Each family consists of three specific isoforms that are composed of ~5000 and ~2700 amino acids for RyRs and IP_3_Rs respectively. IP_3_Rs are found at relatively low expression levels in all cell types, whereas RyRs have a more limited tissue expression and with the highest levels observed in striated muscles. Both ion channels are located primarily on the ER (endoplasmic reticulum) membrane, which in muscle is known as the SR (sarcoplasmic reticulum). Their membrane topology appears to be very similar with the proposed minimum of six membrane-spanning regions clustered together within the C-terminal domain of ~500 residues. Both of these integral endomembrane protein families produce cation-permeable channels with relatively poor selectivity for Ca^2+^, but very large conductance. This is not surprising since the predicted pore-forming sequences, namely the fifth and sixth putative transmembrane regions and the intervening pore-loop comprising the selectivity filter, comprise the areas of highest (~50%) sequence similarity. Functional channels are composed of four identical subunits forming homotetramers, whereas the IP_3_R may also form mixed heterotetramers between its three subtypes.

The oligomerization determinants of RyR and IP_3_R are believed to reside within their C-terminus, involving the membrane-spanning regions and the cytoplasmic C-terminal tail. Recombinant IP_3_R1 lacking the membrane-spanning sequences was reported to be in monomeric form [3,4], whereas an expressed IP_3_R1 C-terminal domain (residues 2216–2749) was able to assemble into tetramers [5]. Further deletion constructs indicated that the primary oligomerization sites lie within the last two transmembrane regions near to the C-terminus, whereas secondary elements are also present within the preceding four transmembrane segments [6,7]. The extreme cytoplasmic C-terminal tail constitutes an additional oligomerization determinant since it is itself capable of self-association and its presence enhanced tetramerization of recombinant IP_3_R1 C-terminal fragments [4,6,8]. Similar results have been reported for RyR; deletion of the C-terminal 15 residues resulted in an inactive RyR1 channel due to impaired tetrameric assembly [9], whereas the isolated RyR2 C-terminal tail (residues 4867–4967) was shown to form tetramers [10].

Between these two protein families, the N-terminus is the region with the second-highest level of sequence similarity (~40%), implying a potentially conserved role in channel function. Indeed, expression of protein chimaeras formed between IP_3_R and RyR indicated that N-terminal sequences of one channel could be functionally substituted by the corresponding region of the other [11]. Accordingly, the 3D structures of the IP_3_R and RyR N-termini obtained from X-ray crystallography are remarkably similar [11,12]. In IP_3_R, the N-terminus forms the IP_3_-binding site, which can be divided into the ligand binding ‘core’ (residues 224–579) and the ‘suppressor domain’ (residues 1–223), with the latter inhibiting IP_3_ binding to the core [13,14]. In RyRs, the N-terminus constitutes one of the three mutation loci associated with ion channel dysfunction leading to arrhythmogenic cardiac disease and neuromuscular disorders [2,15].

We have recently reported that the cardiac RyR2 N-terminus is found in tetrameric form, which helps regulate the closed channel conformation during the diastolic phase of cardiomyocyte contraction [16]. In the present study, we provide evidence that N-terminus tetramerization is a structural feature that is conserved across the three mammalian RyR isoforms and, significantly, this innate ability to oligomerize into tetramers is observed also in the N-terminus of the related IP_3_R1 intracellular Ca^2+^ release channel.

## EXPERIMENTAL

### Materials

Cell culture reagents and high-molecular-mass markers (HiMark) were obtained from Invitrogen (Life Technologies), other electrophoresis reagents were from Bio-Rad Laboratories, protease inhibitor cocktail (Complete) was from Roche, CHAPS and calpain-2 were from Calbiochem (Merck), and IP_3_ and all other reagents were from Sigma.

### Plasmid construction

RyR1/2/3 cDNA for expression of N-terminal domains (BT4LR1/2/3) was generated by PCR amplification using plasmids containing the full-length cDNA sequence of human RyR2 or rabbit RyR1 and RyR3. Primers used were: BT4LR1 [5′-CATGGGTGACGGAGGAGAGGG-3′ (forward) and 5′-AAGACTCGAGCCTCTTGTTGTCATCCCGAACC-3′ (reverse)], BT4LR2 [5′-CGCGGAACCATGGCCGAT-3′ (for-ward) and 5′-TTGGATCCGGACCATACTGCCAGCCA-3′ (rev-erse)] and BT4LR3 [5′-CATGGCCGAAGGCGGAGAAGG-3′ (forward) and 5′-TAGGCTCGAGTTTTGAAAACTCCACCA-3′ (reverse)]. PCR products were cloned into Y2H (yeast two-hybrid) vectors pGBKT7 and pACT2 (Clontech), and mammalian expression vector pCR3 (Invitrogen, Life Technologies) containing c-Myc or HA (haemagglutinin) peptide epitope tags. IP_3_R1 cDNA for expression of the N-terminal domain (residues 1–667) was generated by PCR amplification from a human hippocampus cDNA library (Stratagene, Agilent Technologies) using primers 5′-TGGCGAATTCATGTCTGACAAAATGTC-TAG-3′ (forward) and 5′-TCCAGTCGACGAGACACCTTC-AAATTCAAAA-3′ (reverse), and was then cloned into c-Myc- and HA-modified pCR3 vector. The IP_3_R1^1–667, K493A^ mutant was generated using the QuikChange® XL site-directed mutagenesis kit (Stratagene, Agilent Technologies) and complementary oligonucleotide primers [5′-CCAACAGAGAA-CGGCAGGCACTGATGAGAGAACAG-3′ (forward) and 5′-CT-GTTCTCTCATCAGTGCCTGCCGTTCTCTGTTGG-3′ (rev-erse)]. All plasmids were verified by direct DNA sequencing (BigDye, PerkinElmer).

### Calpain cleavage of native RyR1 and chemical cross-linking

Rabbit skeletal muscle SR (10 μg) was resuspended in buffer [10 mM Na_2_Pipes, 120 mM KCl and 10 mM DTT (pH 7.4)], supplemented with 2 mM CaCl_2_, and incubated with 4 units of calpain-2 for 5 min at room temperature (22°C). SR vesicles were pelleted at 20000 ***g*** for 10 min at 4°C, the supernatant was saved, and the SR pellet was resuspended in fresh buffer. Resuspended SR pellet and supernatant were incubated with 0.0025% glutaraldehyde. The reaction was stopped with 2% hydrazine and samples were analysed by Western blotting using the anti-RyR1 antibody Ab^2142^ (raised against residues 830–845).

### Other methods

The Y2H system, HEK (human embryonic kidney)-293 cell culture and transfection, chemical cross-linking, co-immunoprecipitation, SR preparation and Western blotting analysis were carried out as described previously [16–18]. Densitometry analysis was performed using a GS-700 scanner (Bio-Rad Laboratories) and Quantity One software (Bio-Rad Laboratories). Data are expressed as means±S.E.M. Statistical analysis was performed using an unpaired Student's *t* test.

## RESULTS

### N-terminus tetramerization is conserved in mammalian RyR isoforms

**Figure 1:**
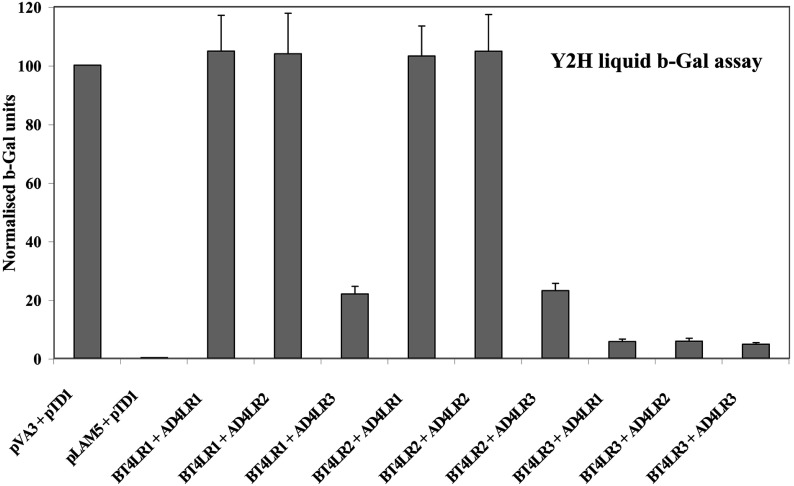
Promiscuous RyR1/2/3 N-terminus interactions in yeast cells Quantitative liquid β-galactosidase assays (*n*=5) of yeast Y190 co-transformed with RyR1/2/3 (residues 1–915, 1–906 and 1–911 respectively) fused with GAL4 DNA-BD (BT4LR1/2/3) or GAL4 AD (AD4LR1/2/3) as indicated. Results are normalized against the positive control pair pVA3+pTD1 (pVA3 encodes GAL4 DNA-BD fusion with p53 protein; pTD1 encodes GAL4 AD fusion with the SV40 large T antigen).

We have recently used a complementary series of Y2H, chemical cross-linking and co-immunoprecipitation assays to demonstrate that an RyR2 N-terminal fragment (residues 1–906) is capable of self-association, leading to tetramer assembly [16]. In the present study, we investigated whether the corresponding N-terminal fragments from the disparate isoforms, RyR1 (residues 1–915) and RyR3 (residues 1–911), are also capable of self-interaction to form oligomers. Hence, Y2H protein interaction analysis was carried out with the yeast strain Y190, co-transformed with various RyR isoform fragments fused to both the GAL4 DNA-BD (DNA-binding domain) (BT4LR1/2/3) and GAL4 AD (activation domain) (AD4LR1/2/3). Quantitative β-galactosidase assays indicated a very strong interaction for the BT4LR2/AD4LR2 pair (Figure 1), as has been reported previously [16]. Robust interactions were similarly found for the RyR1 N-terminus fragments (BT4LR1+AD4LR1) and, notably, also for the RyR1–RyR2 mixed pairs (Figure 1). The RyR3 N-terminus self-interaction, as well as interaction between the mixed isoform pairs RyR1–RyR3 and RyR2–RyR3, was also detected in β-galactosidase assays, albeit considerably weaker than those for RyR1 and RyR2. These findings suggest that N-terminus self-association is observed in isoforms RyR1 and RyR3 and it is therefore not unique to the cardiac RyR2 isoform.

**Figure 2:**
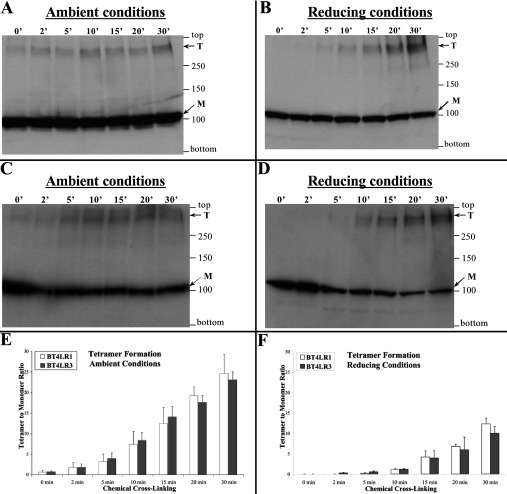
A tetramer is the predominant oligomeric form of the RyR1/3 N-terminal domain Chemical cross-linking assays from HEK-293 cell homogenates expressing BT4LR1 (RyR1 residues 1–915) (**A** and **B**) or BT4LR3 (RyR3 residues 1–911) (**C** and **D**). Cell homogenate, pre-treated without (**A** and **C**) or with (**B** and **D**) 10 mM DTT, was incubated with glutaraldehyde for the indicated time points and analysed by SDS/PAGE (6% gels) and Western blotting using an anti-c-Myc antibody. The proteins corresponding to the monomeric (M, ~100 kDa) and tetrameric (T) forms are indicated by the arrows. (**E** and **F**) Densitometry analysis (*n*=3) was carried out on the bands corresponding to the tetramer and monomer and used to express tetramer/monomer abundance.

To examine the precise stoichiometry of the RyR1/3 N-terminus oligomer, we expressed RyR1/3 N-terminal fragments tagged with the c-Myc peptide epitope (BT4LR1/3) in mammalian HEK-293 cells and carried out chemical cross-linking using glutaraldehyde. Western blot analysis demonstrated the existence of two anti-c-Myc immunoreactive protein bands: the ~100 kDa monomer and a tetrameric species that increased in a time-dependent manner for both RyR1 and RyR3 N-termini following the addition of glutaraldehyde (Figure 2). Notably, minimal dimer and no trimer bands were detected, as was also seen with the corresponding RyR2 N-terminal fragment [16]. Although it is a minor component, the tetrameric form is evident in ambient conditions even before the addition of glutaraldehyde (0’ time point), but this was abolished by pre-treatment with the reducing agent DTT (0’ time point, 10 mM DTT) (Figures 2B and 2D). This indicates that a small proportion of the expressed RyR1/3 N-terminal domain already exists as a disulfide-linked tetramer in HEK-293 cells. Quantification (*n*=3) of the anti-c-Myc immunoreactive proteins using densitometry analysis (Figures 2E and 2F) demonstrated that RyR1 and RyR3 N-termini form tetramers to a similar extent when expressed in mammalian cells.

**Figure 3:**
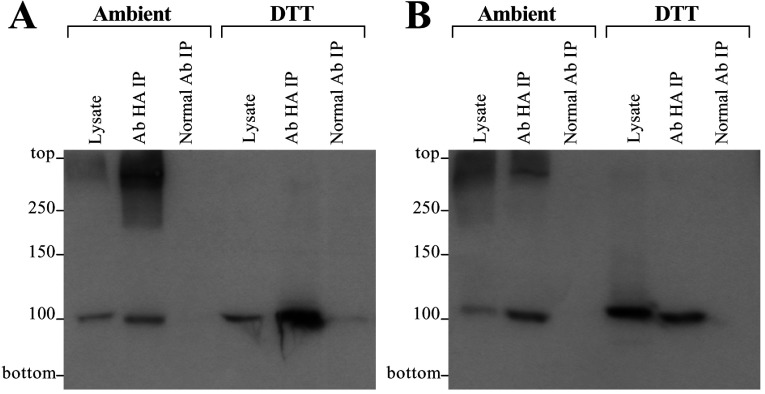
RyR1/2/3 N-terminal fragments form mixed heterotetramers in mammalian cells Co-immunoprecipitation assays from HEK-293 cell lysates co-expressing HA-tagged RyR2 residues 1–906 (AD4LR2) and c-Myc-tagged RyR1 residues 1–915 (BT4LR1) (**A**) or RyR3 residues 1–911 (BT4LR3) (**B**). AD4LR2 was immunoprecipitated (IP) with an anti-HA antibody from CHAPS-solubilized HEK-293 cell lysates, pre-treated with or without 10 mM DTT as indicated, and the presence of associated BT4LR1/3 was analysed by SDS/PAGE (6% gels) and Western blotting using an anti-c-Myc antibody. Cell lysate, 1/50th of the volume processed in immunoprecipitation samples, was also included to serve as a molecular mass standard. Representative blots from three experiments are shown. Ab, antibody.

To aid in validation of the Y2H observations that RyR1/2/3 N-terminal fragments interact with each other, we performed co-immunoprecipitation assays from HEK-293 cells co-expressing HA-tagged RyR2 N-terminus (residues 1–906, AD4LR2) and c-Myc-tagged RyR1/3 N-termini (residues 1–915 and 1–911 respectively; BT4LR1/3). HA–AD4LR2 from CHAPS-solubilized HEK-293 cell lysate was immunoprecipitated with an anti-HA antibody and the presence of co-precipitated c-Myc–BT4LR1/3 was analysed by Western blotting using an anti-c-Myc antibody. The c-Myc-tagged BT4LR1 (~100 kDa) was specifically recovered only in the anti-HA immunoprecipitation, not in the control immunoprecipitation with normal non-immune serum (Figure 3A). In ambient conditions, an additional anti-c-Myc immunoreactive high-molecular-mass band was also detected in the anti-HA immunoprecipitation, suggesting that a mixed oligomer had formed comprising BT4LR1 and AD4LR2. This hetero-oligomeric species was abolished by treatment with the reducing agent DTT, suggesting the existence of disulfide bonding between the RyR1/2 protomers (Figure 3A). Similar co-immunoprecipitation results were obtained for the AD4LR2/BT4LR3 pair (Figure 3B). These findings suggest that the recombinant N-terminal domains of RyR1/2/3 are able to interact with each other and they also form disulfide-linked heterotetramers when co-expressed in mammalian cells.

**Figure 4:**
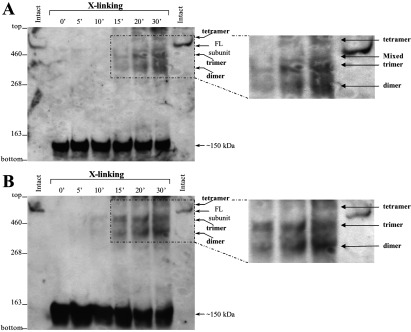
Native RyR1 N-terminus forms oligomers Chemical cross-linking assays of calpain-cleaved RyR1. Calpain cleavage of rabbit skeletal muscle SR pre-treated with 10 mM DTT, followed by centrifugation to recover SR vesicles. Resuspended SR pellet (**A**) and supernatant (**B**) were incubated with glutaraldehyde for the indicated time points and analysed by SDS/PAGE (4% gels) and Western blotting using Ab^2142^ against the RyR1 N-terminus. Intact RyR1 was also included to serve as a molecular mass standard. Intact full-length (FL) or ‘reconstituted’ RyR1 subunit, calpain-cleaved ~150 kDa N-terminal fragment and oligomeric forms are shown with the arrows. Representative blots from three experiments are shown; part of the blot is enlarged for the minor bands corresponding to tetramer and mixed N-terminus plus C-terminus cross-linked species to be more clearly visible.

We further investigated the oligomeric state of the N-terminus in the native RyR1 from rabbit skeletal muscle SR. Native RyR1 was incubated with the calpain protease to generate the characteristic cleavage into ~150 kDa N-terminal and ~400 kDa C-terminal proteolytic fragments, followed by centrifugation to recover SR vesicles. The resuspended SR pellet contained the proteolysed RyR1 C-terminal fragment and a proportion of the N-terminus that remained associated with the transmembrane assembly, whereas the supernatant contained only the liberated N-terminal fragment. Chemical cross-linking using glutaraldehyde was carried out on both the SR pellet and the supernatant, and calpain-cleaved RyR1 was monitored by Western blotting using an N-terminal-specific anti-RyR antibody (Ab^2142^). For the SR membranes, in addition to the 150 kDa N-terminal protomer, we observed time-dependent formation of two major bands of ~300 kDa and ~450 kDa, which correspond to a dimer and trimer of the 150 kDa fragment respectively (Figure 4A). Two minor bands of larger mass were also weakly detected: the first minor band, migrating just above the full-length RyR1 subunit at ~600 kDa, is attributable to the tetramer (of 150 kDa); the second minor band, with relative mobility equivalent to the full-length RyR subunit (560 kDa), is consistent with a ‘reconstituted’ N-terminus plus C-terminus (i.e. ~150+~400 kDa) cross-linked species. We note that although the two minor bands were reproducibly observed, they are of very low abundance. Importantly, similar results were obtained following chemical cross-linking of the supernatant (Figure 4B), which contains only the calpain-cleaved RyR1 N-terminal fragment that has dissociated from the transmembrane assembly (which remains with the SR pellet). Both the dimer and trimer oligomeric species of the 150 kDa N-terminal fragment were again readily detected (~300 kDa and ~450 kDa respectively); moreover, a minor band of larger mass at ~600 kDa corresponding to the tetramer could also be seen, but there was no evidence for the mixed N-terminus plus C-terminus cross-linked species of 560 kDa (Figure 4B). These results indicate that the RyR1 N-terminus exists in a tetrameric form in the native protein and that this specific oligomeric association is retained even after calpain proteolytic cleavage.

### N-terminus tetramerization is conserved in IP_3_R

**Figure 5:**
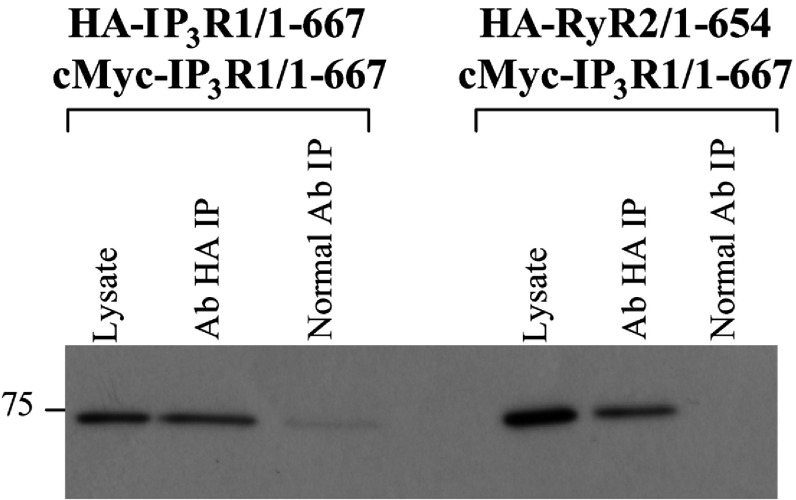
IP_3_R1 N-terminus domain self-interaction in mammalian cells Co-immunoprecipitation assays from HEK-293 cell lysates co-expressing c-Myc-tagged IP_3_R1 residues 1–667 and HA-tagged IP_3_R1 residues 1–667 or RyR2 residues 1–654 as indicated. HA-tagged proteins were immunoprecipitated (IP) with an anti-HA antibody from CHAPS-solubilized HEK-293 lysates, pre-treated with 10 mM DTT, and the presence of associated c-Myc–IP_3_R1^1–667^ was analysed by SDS/PAGE (6% gel) and Western blotting using an anti-c-Myc antibody. Cell lysate, 1/50th of the volume processed in immunoprecipation samples, was also included to serve as a molecular mass standard. A representative blot from three experiments is shown. Ab, antibody.

Given the high degree of sequence homology and structural similarity between RyR and IP_3_R N-termini, it is plausible that the latter may also be capable of self-association. In order to test the possibility of IP_3_R N-terminus oligomerization, we generated an IP_3_R1 fragment comprising residues 1–667 (human IP_3_R1/SI^−^ co-ordinates) based on sequence similarity with RyR N-terminal domains (~40% similarity between IP_3_R1 residues 117–656 and RyR2 residues 115–649) and also on our observations that a truncated RyR2 N-terminal fragment (residues 1–654) retains the ability to form tetramers (M. Seidel and S. Zissimopoulos, unpublished work). The IP_3_R1 N-terminus self-interaction, as well as any potential oligomeric interaction with the homologous RyR2 domain, was then assessed by co-immunoprecipitation assays. The HA-tagged IP_3_R1^1–667^ or HA-tagged RyR2^1–654^ was co-expressed with c-Myc-tagged IP_3_R1^1–667^ in HEK-293 cells. Immunoprecipitation of HA–IP_3_R1^1–667^ with an anti-HA antibody resulted in co-precipitation of c-Myc–IP_3_R1^1–667^, whereas no interaction was detected in the control immunoprecipitation with normal non-immune antibody (Figure 5). Interestingly, immunoprecipitation of HA–RyR2^1–654^ with an anti-HA antibody also indicated evidence for the co-precipitation of c-Myc–IP_3_R1^1–667^ (Figure 5), suggesting that the discrete IP_3_R1 and RyR2 N-termini have the potential to form hybrid oligomers.

**Figure 6:**
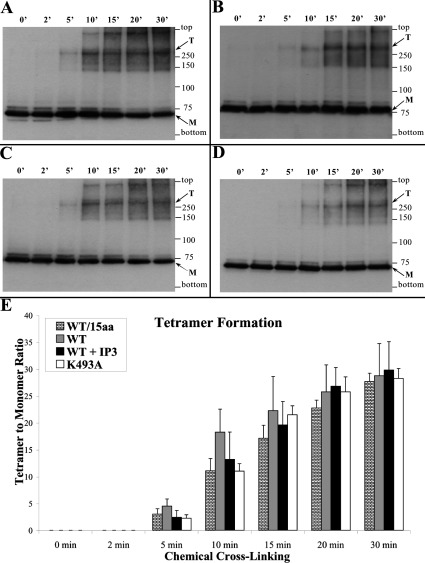
The IP_3_R1 N-terminus forms tetramers in an IP_3_-independent manner Chemical cross-linking assays from HEK-293 cell homogenates expressing either wild-type IP_3_R1 residues 1–667 pre-treated without (**A**) or with (**C**) 10 μM IP_3_, or expressing IP_3_R1/SI^+1–667^ (containing the 15-residue splice insert) (**B**), or mutant IP_3_-binding deficient IP_3_R1^1–667, K493A^ (**D**). Cell homogenates, pre-treated with 10 mM DTT, were incubated with glutaraldehyde for the indicated time points and analysed by SDS/PAGE (6% gels) and Western blotting using an anti-c-Myc antibody. The proteins corresponding to monomeric (M, ~73 kDa) and tetrameric (T) forms are indicated by the arrows; the mobility of the high-molecular-mass band close to the top of the gel is consistent with a mixed cross-linked species between the recombinant N-terminal fragment and full-length endogenous HEK-293 IP_3_R. (**E**) Densitometry analysis (*n*=4) was carried out on the bands corresponding to the tetramer and monomer and used to express tetramer/monomer abundance; there was no statistical significance (*P*>0.1) between samples for any given cross-linking time point.

In chemical cross-linking experiments, the c-Myc-tagged IP_3_R1^1–667^ (~73 kDa) was expressed in HEK-293 cells, then a reaction with glutaraldehyde was carried out under reducing conditions and evidence for oligomer formation was monitored by Western blotting using an anti-c-Myc antibody. We observed the presence of time-dependent formation of protein bands corresponding to a dimer, trimer and tetramer, with the tetramer band being the predominant oligomeric species (Figure 6A). Similar results were obtained for IP_3_R1/SI^+1–667^ containing the splice insertion (Figure 6B), suggesting that the presence of the 15-residue splice insertion (at Asp^321^) does not affect the IP_3_R1 N-terminus oligomerization.

**Figure 7:**
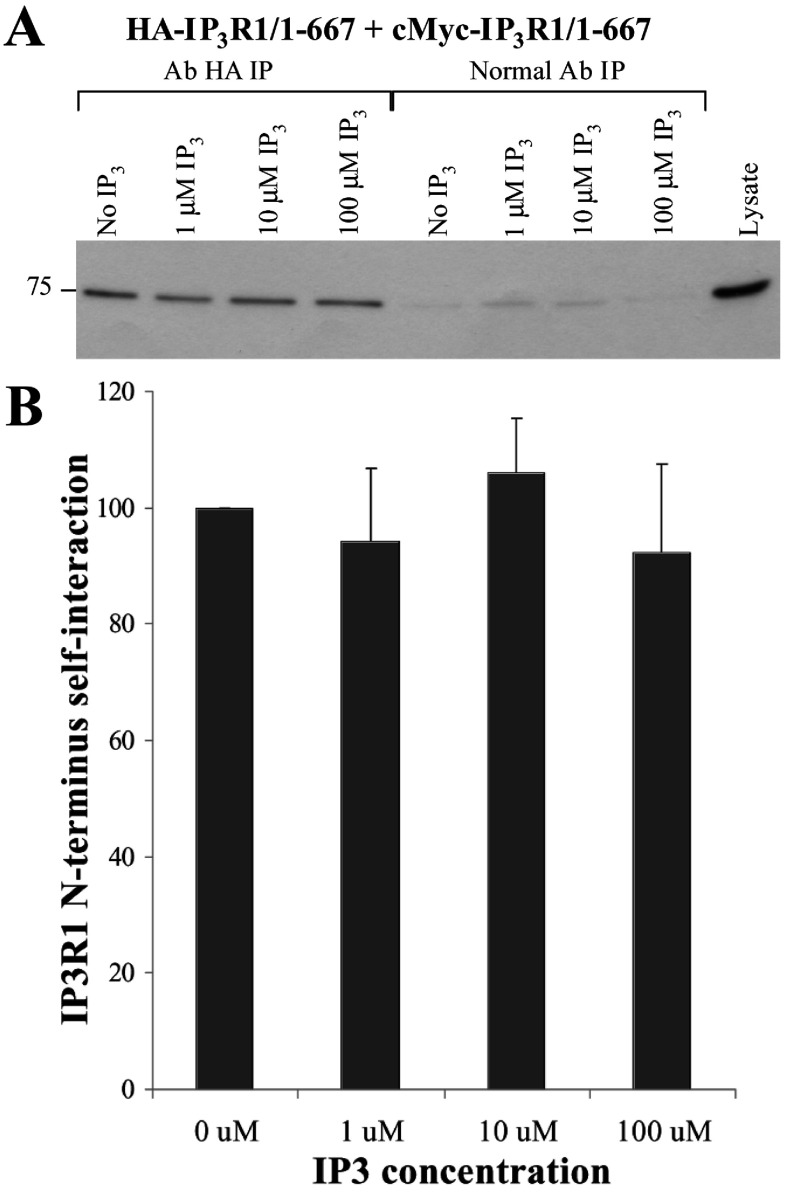
IP_3_-independent self-interaction of the IP_3_R1 N-terminus (**A**) Co-immunoprecipitation assays from HEK-293 cell lysates co-expressing c-Myc- and HA-tagged IP_3_R1^1–667^ was carried out in the presence of various IP_3_ concentrations as indicated. HA–IP_3_R1^1–667^ was immunoprecipitated with an anti-HA antibody from CHAPS-solubilized HEK-293 lysates, pre-treated with 10 mM DTT and supplemented with IP_3_ as shown, and the presence of associated c-Myc–IP_3_R1^1–667^ was analysed by SDS/PAGE (6% gel) and Western blotting using an anti-c-Myc antibody. Cell lysate, 1/50th of the volume processed in immunoprecipitation samples, was also included to serve as a molecular mass standard. (**B**) Densitometry analysis (*n*=4) was carried out on the band corresponding to co-precipitated c-Myc–IP_3_R1^1–667^. Data are normalized for the signal obtained in the absence of IP_3_; there was no statistical significance (*P*>0.5) between samples for any of the IP_3_ concentrations tested.

To examine for any effect of the addition of IP_3_ ligand on IP_3_R1 N-terminus oligomerization, we conducted chemical cross-linking experiments on HEK-293 cell homogenates expressing c-Myc–IP_3_R1^1–667^. Experiments using either 1 or 10 μM IP_3_ treatment before chemical cross-linking had a minimal effect on tetramer formation (Figures 6A and 6C). Indeed, cumulative data following densitometry analysis (*n*=4) indicate that the presence of IP_3_ has no statistically significant effect on IP_3_R1^1–667^ tetramerization (Figure 6E). To test for the possibility that the recombinant IP_3_R1 N-terminal fragment expressed in HEK-293 cells has been saturated by basal IP_3_ levels, we generated a construct carrying the K493A mutation, previously shown to abolish IP_3_ binding [14]. The IP_3_ binding-deficient mutant, IP_3_R1^1–667, K493A^, was found to form tetramers to the same extent as the wild-type IP_3_R1^1–667^ (Figures 6D and 6E), arguing against the possibility of endogenous HEK-293 IP_3_ levels directly affecting N-terminus tetramerization. The effect of IP_3_ on IP_3_R1^1–667^ self-interaction was also tested by co-immunoprecipitation assays. We found that the amount of c-Myc–IP_3_R1^1–667^ recovered with immunoprecipitated HA–IP_3_R1^1–667^ was not affected by IP_3_ concentrations up to 100 μM (Figure 7A). Quantification (*n*=4) using densitometry analysis (Figure 7B) demonstrated no statistically significant effect for any of the IP_3_ concentrations on IP_3_R1 N-terminus self-association.

## DISCUSSION

We have identified that a novel oligomeric interaction exists between the N-terminal regions of distinct subunits that form tetrameric RyR and IP_3_R intracellular Ca^2+^ release channels. A complementary combination of Y2H, co-immunoprecipitation and chemical cross-linking assays collectively demonstrate that the discrete N-terminal domain of all three mammalian RyRs can intrinsically assemble into homo- and hetero-tetramers (Figures 1–3). Importantly, the calpain-cleaved N-terminus of native skeletal muscle RyR1 was shown to exist in oligomeric form, although the tetrameric species was of very much lower abundance than that of the dimer and trimer, possibly due to inherent instability of the tetramer following proteolytic cleavage away from the transmembrane domain (Figure 4). Similarly, the type 1 IP_3_R N-terminal domain is able to tetramerically associate with itself, as well as with the homologous RyR2 N-terminal fragment to form IP_3_R–RyR hybrid oligomers (Figures 5 and 6). The presence of either a 15-residue splice insertion, or of the cognate ligand IP_3_ at micromolar concentrations did not affect IP_3_R1 N-terminus tetramerization (Figures 6 and 7). These combined empirical findings for both RyR and IP_3_R indicate that N-terminus self-association may be a fundamental feature of this region that is conserved across isoforms and applies to both of these families of intracellular Ca^2+^ release channels.

The observed RyR1/3 N-terminus tetramerization is consistent with our previous findings for the cardiac isoform RyR2 [16], and indicates that this important oligomerization determinant may be an intrinsic property of the native N-terminal domain in all mammalian RyRs. It was previously reported that truncated RyR1 without the C-terminal 15 amino acids lacks high-affinity ryanodine binding that was attributed to impaired tetrameric assembly [9], although it is possible that the deletion could have directly affected the ryanodine-binding site. Our findings suggest that N-terminus self-association could play a role in the formation of full-length RyR tetrameric channels, in addition to other oligomerization determinants, including the C-terminal region [9,10]. Tetramerization of the recombinant RyR N-terminal domain is primarily mediated by non-covalent protein–protein interactions, which may be further stabilized by covalent disulfide bonds (Figures 2 and 3). Disulfide-linked N-terminus tetramers were previously observed in native RyR2 from pig heart, but not in native RyR1 from rabbit skeletal muscle [16]. Despite the absence of disulfide bonds, the N-terminal domain (~150 kDa calpain-cleaved fragment) within the full-length native RyR1 interacts with itself and forms oligomers (Figure 4). Notably, the predominant protein species obtained from chemical cross-linking of calpain-cleaved native RyR1 corresponds to N-terminal oligomers with minor amounts of the mixed N-terminal/C-terminal species (Figure 4A). Hence, N-terminal/C-terminal interactions appear secondary in relative potency compared with the intersubunit N-terminus self-association evident within native RyR1. The RyR N-terminus self-association appears to be somewhat promiscuous since recombinant N-terminal fragments from all three mammalian isoforms were found to interact with each other (Figures 1 and 3). This finding is consistent with the high sequence homology between RyR isoforms (~85% for the first 900 residues); however, its physiological significance is unclear. Although recombinant RyR2 has been shown to form heteromeric channels with RyR1 and RyR3 when heterologously co-expressed in HEK-293 cells [19], there is no evidence to date for the existence of RyR heterotetramers in native tissue.

**Figure 8:**
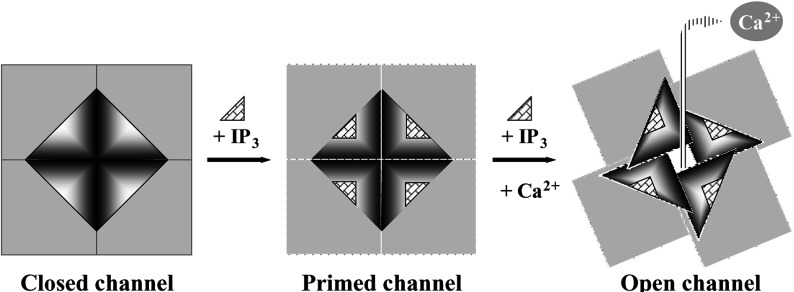
Proposed model for N-terminus tetramerization in IP_3_R1 channel conformation Schematic representation of the IP_3_R1 N-terminal domain (triangle) that interacts with adjacent N-terminal domains to form a tetramer that surrounds the central four-fold axis, and a proposed co-operative mode of action for the activating ligands IP_3_ and Ca^2+^. IP_3_ binding alone does not alter N-terminal intersubunit interactions but primes IP_3_R1 for activation. Concerted binding of IP_3_ and Ca^2+^ results in a modified N-terminus oligomeric interaction leading to channel opening.

Similar to the three RyR isoforms, we found that the discrete N-terminus of IP_3_R1 (residues 1–667) can also readily oligomerize (Figures 5 and 6). This finding appears inconsistent with two earlier reports demonstrating that truncated IP_3_R1 lacking the C-terminal transmembrane domain remains in a monomeric form [3,4]. Methodological differences are likely to account for the discrepant results, e.g. sucrose-density-gradient centrifugation of solubilized proteins was performed using a high concentration (0.9%) of the zwitterionic detergent CHAPS [3], or the use of different expression systems and chemical cross-linking reagents [4]. In contrast, our results are consistent with, and importantly provides direct empirical evidence to support, the recently proposed N-terminus location within the IP_3_R1 3D architecture [11]. Docking the crystal structure of the IP_3_R1 N-terminus (residues 1–604) into the low-resolution map of native IP_3_R1 revealed an arrangement where the N-termini are placed proximal to each other in a closed circular tetrameric fashion [11]. Moreover, two very recent reports concur with our present study, providing evidence for IP_3_R1 N-terminus oligomerization from bead aggregation and chemical cross-linking assays [20,21]. Indirect evidence is also provided by FRET measurements of two fluorescent proteins fused to the N-terminus of individual IP_3_R1 subunits, suggesting that the N-termini from adjacent subunits are in physical proximity [22]. Importantly, we have observed that IP_3_R N-terminus tetramerization appears to be independent of IP_3_ at micromolar levels (Figures 6 and 7). In light of our previous findings which suggest that destabilization of N-terminal intersubunit interactions results in RyR2 activation [16], and since IP_3_ binding is a prerequisite for IP_3_R channel opening, we hypothesized that IP_3_R activation by IP_3_ may be mediated by a similar molecular mechanism involving altered N-terminal interactions, a hypothesis that was also proposed by other research groups [11]. Very recently, it was shown that unusually high (non-physiological) IP_3_ concentrations (1.5 mM) result in reduced tetramerization of a mutant cysteine-less IP_3_R1 N-terminal fragment purified from bacteria [21]. However, our chemical cross-linking and co-immunoprecipitation assays on IP_3_R1 N-terminus expressed in mammalian HEK-293 cells demonstrates that IP_3_ up to 100 μM had no effect on N-terminus tetramerization (Figures 6 and 7). We should point out that in the aforementioned study [21], lower IP_3_ concentrations (50 μM) that are closer to the physiological range were also found to be ineffective, in agreement with our results. Thus it is apparent that physiological levels of IP_3_ are unlikely to disrupt N-terminal intersubunit interactions, at least for the isolated recombinant IP_3_R1 N-terminus domain. On the basis of this empirical evidence, we propose that IP_3_ binding may prime the IP_3_R1 for channel opening without affecting N-terminus oligomerization (Figure 8). This is consistent with the inability of IP_3_ alone to activate IP_3_R1, with channel opening elicited specifically by the concerted action of IP_3_ and Ca^2+^ binding [1,23]. When both IP_3_ and Ca^2+^ co-agonists are bound, N-terminal intersubunit interactions are altered resulting in IP_3_R1 channel opening (Figure 8). Whether the effect of Ca^2+^ on N-terminus tetramerization is direct, due to binding at the proposed Ca^2+^-binding sites within the N-terminus (residues 304–450) [24,25], or allosteric, remains to be elucidated.

RyRs and IP_3_Rs are related families of intracellular Ca^2+^ channels that are likely to have evolved from a common ancestor [26,27]. They both form very large tetrameric protein complexes that have similar biophysical properties, membrane topology and pore structure. N-terminus tetramerization is another important structure–function trait that we now show has been conserved through the evolution of these channels. N-terminal intersubunit interactions have been implicated in the molecular mechanism controlling channel closure for both channel families [11,16,21,28]. However, it should also be noted that RyRs and IP_3_Rs exhibit significant functional differences regarding the role of the N-terminus in channel regulation. First RyRs are not activated by IP_3_ and there is currently no evidence that its N-terminus binds this or any other surrogate ligand. Secondly, although deletion of the suppressor domain (residues 1–223) results in unresponsive IP_3_R1 channels [13,29], deletion of the first 305 residues in RyR2 apparently has no major adverse effects on channel function [28,30]. Further studies should help to unravel the precise molecular mechanism(s) by which RyR/IP_3_R N-terminus oligomerization is involved in the dynamic isoform-specific regulation of intracellular Ca^2+^ release.
